# Vocational training for unemployed youth in Latvia

**DOI:** 10.1007/s00148-021-00877-8

**Published:** 2021-11-18

**Authors:** Massimiliano Bratti, Corinna Ghirelli, Enkelejda Havari, Giulia Santangelo

**Affiliations:** 1 Universitá degli Studi di Milano, Milan, Italy; 2 Centro Studi Luca d’Agliano, LdA, Milan, Italy; 3 Global Labor Organization (GLO) gGmbH GLO, Leimkugelstr. 6, 45141 Essen, Germany; 4 Institute of Labor Economics, IZA, Bonn, Germany; 5 Bank of Spain, Directorate General Economics, Statistics and Research, Madrid, Spain; 6 European Commission, Joint Research Centre, Ispra, Italy; 7 IESEG School of Management, 3 rue de la Digue, 59000 Lille, France

**Keywords:** Youth guarantee, Youth unemployment, Vocational training, Impact evaluation, J01, J08, J18, J24

## Abstract

We analyze the effectiveness of a vocational training (VT) programme targeting unemployed youth in Latvia, contributing to the scant literature on active labour market policies in transition countries. The programme we analyse is part of the Youth Guarantee scheme (2014–2020), the largest action launched by the European Union to combat youth unemployment after the 2008 financial crisis. Although the programme was targeted to youths aged between 15 and 29, priority was given to those younger than 25 years of age. We exploit this eligibility rule in a fuzzy regression discontinuity design framework to estimate the impact of VT participation on the probability of being employed and gross monthly labour income at given dates after the training. Using rich administrative data, we find that the age priority rule increased programme participation for the youngest group by about 10 percentage points. However, participation in the programme did not lead to statistically significant positive effects in labour market outcomes. We argue that this result could be due to some specific characteristics of the programme, namely the voucher system (potentially inducing lock-in effects) and the type of training (classroom instead of on-the-job training). Moreover, the programme was targeted at ex-ante low-employable individuals (e.g. without vocational qualifications), a fact that is confirmed by our analysis of the characteristics of the population of compliers with the age priority rule.

## Introduction

The global financial crisis led to an increase in unemployment rates across European countries, particularly among young people. In 2013, the average youth unemployment rate in Europe was 23.9% and exceeded 50% in countries such as Greece (58.3%) and Spain (55.5%).^1^ In response, the European Union (EU) launched two actions aiming to tackle youth unemployment: the Youth Employment Initiative (YEI) and the Youth Guarantee (YG). Under the YG initiative, all EU member states committed to implementing policies aimed at reducing youth unemployment.^2^ These plans include a number of active labour market policies (ALMPs)—e.g. apprenticeships, traineeships, job placements, and further education leading to a qualification, which are funded by different EU schemes and national resources. The YEI is one of the main financial instruments supporting the implementation of YG schemes, with a total budget of 8.8 billion euros for the period of 2014–2020. It was mainly targeted at regions with particularly high levels of youth unemployment.^3^

Although the YG plans and national experiences are well documented (Cabasés Piqué et al. 2016; Pastore 2015; Escudero and Mourelo 2015; European Commission 2016), among others), there is scant evidence on the effectiveness of ALMPs implemented through this initiative. The main reason is that most of these programmes are still running and the data collection process for performing rigorous impact evaluations is often incomplete.^4^

Since the YG represents the EU strategy to tackle high unemployment rates among young people, the programme is key to assessing its effectiveness. The aim of this study is to start filling this gap by providing evidence from the recent implementation of the YG scheme in Latvia.

Evaluating YG schemes has general policy relevance since similar programmes are frequently implemented by governments after recessions and downturns. In addition, Latvia provides an interesting case as it was one of the European countries most affected by the 2008 financial crisis, with total unemployment reaching 17.5% by 2009 (33% for youth unemployment)—the highest in the EU after Spain—and the country’s real gross domestic product (GDP) falling by 13% compared to the previous year. As stated by Paul Krugman, ‘The most

acute problems are on Europe’s periphery, where many smaller economies are experiencing crises strongly reminiscent of past crises in Latin America and Asia: Latvia is the new Argentina’ (The New York Times, 2008). Nevertheless, the country showed the fastest recovery in the post-recession period, and Latvia’s GDP has shown stable growth since 2011. Despite these positive results, the youth unemployment rate remained above 25%, making Latvia the ideal candidate country for accessing the YEI funds to finance the national YG plan.

In our analysis, we focus on the evaluation of a large vocational training (VT) programme targeted at unemployed youths aged between 15 and 29 years.^5^ We use a fuzzy regression discontinuity design (FRDD) leveraging a specific eligibility criterion used by the Latvian government that gives higher priority for participation to unemployed people under the age of 25. Presumably exogenous variation induced by the priority rule is leveraged to estimate the causal effect of participation in the VT programme on labour market outcomes (up to 2 years after participation), allowing us to address the issue of individual selection into the VT programme.

We use rich administrative data provided by the Latvian Ministry of Finance. The data contain information on the population of registered unemployed at specific dates (including both participants and non-participants in the VT programme) collected by the Latvian Public Employment Service (known as the State Employment Agency, SEA hereafter), which are matched with data from the State Revenue Service (SRS) that include information on labour income at specific dates before and after participation in the programme.

Our results show that the priority rule significantly predicts participation in the VT programme. Our first-stage results are strong and statistically significant. We find that being younger than 25 years of age increases the likelihood of participation by about 10 percentage points (pp, hereafter). However, we find no statistically significant effect of the training programme on the probability of being employed and on gross monthly income at given calendar dates after training. We argue that this result could be due to some specific characteristics of the programme—namely the voucher system—potentially inducing lock-in effects, and the classroom training, which is generally found in the literature to be less effective than on-the-job training. Moreover, the programme was targeted at ex ante low-employable individuals, a fact that is confirmed by our analysis of the characteristics of the population compliant with the priority rule.

This paper contributes to the rich literature on the evaluation of ALMPs and, more specifically, to the narrower literature on programmes targeting young unemployed persons. First, to our knowledge, this is one of the first papers featuring an evaluation of the YG scheme in Europe for the programme period of 2014–2020.^6^

Second, we provide evidence on the effects of VT in the short and medium terms, as individuals are followed up to 2 years after their participation in the programme. This is an important value added as most of the literature that analyses the effects of large ALMPs on youths focuses on short-term effects. Third, we also provide some characterization for the group of compliers, which may further explain the meagre effectiveness of the programme.

The paper is organised as follows. Section 2 summarises the relevant literature. Section 3 describes the programme and the phases of its implementation. Section 4 presents the data, sample selection, and descriptive statistics. Section 5 explains the empirical strategy, identifying assumptions, and relevant empirical tests for the FRDD. Section 6 discusses the main results and robustness checks, and Section 7 draws conclusions.

## Literature review

The literature on the effects of ALMPs on employment outcomes is very extensive. For brevity, we focus on the extant evidence related to two key features of the programme we are evaluating, namely, the fact that it is targeted at youths and is based on a voucher system.

Several review articles investigate the effectiveness of ALMPs for various countries and population subgroups: see, among others, Martin and Grubb (2001) and Heckman et al. (1999) for the USA and Europe; Kluve (2010), and Card et al. (2010), Caliendo and Schmidl (2016), and Lechner and Wiehler (2011) for different European countries. In particular, Card et al. (2010) carry out a meta-analysis of the effectiveness of ALMPs in many European countries, as well as the USA, Canada, and other anglophone countries. The authors highlight that programmes targeted at young people are generally less effective in improving employment prospects than programmes targeted at adults. This result is confirmed by Kluve (2010) in a study of several European countries: youth training programmes (at least in developed countries) show low-to-modest effects on employment rates.

Card et al. (2018) extend the analysis in Card et al. (2010) by looking at heterogeneous effects of ALMPs by content, target group, state of the business cycle, and by considering a larger sample of studies. They consolidate the previous findings, which can be summarised as follows. First, ALMPs tend to be more effective for the long-term unemployed. Second, training programmes and programmes aimed at increasing an individual’s human capital are more likely to be effective, especially in the long run, compared to job-search assistance programmes. Third, effects tend to be smaller for youths than for adults (consistent with the fact that the effects are larger for the long-term unemployed). Additionally, due to the presence of the so-called lock-in effects, participants in training programmes may reduce their job search efforts and devote more time to programme activities. This implies that the measured positive effects appear to be larger in the long run (2+ years after the end of the programme, according to the authors’ definition) and medium run (1–2 years post-programme) than in the short run (less than 1 year after the programme). Finally, ALMPs are more effective in periods of economic downturn.

Nevertheless, the empirical literature also provides evidence of specific cases where ALMPs have positive impacts. For Europe, for instance, Lechner and Wiehler (2011) document positive employment effects of ALMPs in Austria for young women.^7^ For the USA, Job Corps stands out as one of the few large-scale programmes of (residential) vocational education and training with a positive impact. Launched in 1964 to target disadvantaged youths in the 16–24 age group, it showed positive post-programme earning gains for older youths (Schochet et al. 2008), and especially for white and black individuals but not Hispanics (Flores-Lagunes et al. 2010), with much more pronounced differences for males, whites, and those aged 20–24 at the upper quantiles of the earnings distribution (Eren and Ozbeklik 2014).^8^ The empirical literature also provides various examples of evaluations of youth-targeted labour market policies with clear eligibility criteria based on age, exploited using regression discontinuity designs (RDDs). For instance, Cockx and Dejemeppe (2012) evaluate a Belgian reform that imposed job search requirements only on long-term unemployed benefit claimants younger than 30. Picchio and Staffolani (2019) consider the same age threshold of 30 to analyse whether apprenticeship contracts, which can be stipulated up to 30 years, are more effective pathways into permanent jobs compared to other forms of temporary jobs in Italy. Cockx and Van Belle (2019) exploit two policy discontinuities at 26 and 25 years of age to evaluate the impact on employment of two Flemish active labour market policies for youths. In all cases above, since the assignment to the treatment is partly determined by age, the RDD is of the fuzzy type. The first one finds that the reform enhanced the transition into employment but not into training; Picchio and Staffolani (2019) show the positive impact of apprenticeships, especially within the same firm, while Cockx and Van Belle (2019) document that only the Youth Work Plan positively affected the job-finding rate but that both policies lowered the number of working days, resulting in lower earnings. Given the assumptions of RDDs, all of these studies discuss the external validity of the results. In particular, if one cannot assume that the identified local effect is homogeneous across individuals, thus this cannot be extrapolated for younger or older people further away from the age cut-off.^9^ Moreover, as it has emerged from the studies mentioned above, the statistical power of the RDD and the precision of the estimates depend on the size of the population close to the age thresholds, and hence on the width of the age range considered.

### Evidence on programmes similar to the YG scheme

Programmes similar to the YG were implemented in the Nordic countries and the UK in the 1980s and 1990s, including the British New Deal for Young People (NDYP), the Danish Youth Unemployment Program (YUP), and the German *Jugend mit Perspektive* (JUMP). Sweden was the first country to introduce its own programme for young people in 1984, followed by Norway in 1993 and Denmark and Finland in 1996 (Mascherini 2012; Escudero and Mourelo 2015). Similarly, the UK implemented the NDYP in 1998 to target unemployed young people aged 18–24.

A common feature of these programmes is a strict age limit on participation. In the case of Sweden, Carling and Larsson (2005) analysed a guarantee for early programme participation introduced in 1998 that was similar in spirit to the YG: it targeted unemployed individuals aged 20–24 and aimed to prevent long-term unemployment by guaranteeing assignment to one of the ALMPs within the first 100 days of unemployment. They show that the workplace practice and training programmes had a positive effect on youth employability in the short term but no impact in the long term. As for the UK, Blundell et al. (2004) analyse the NDYP programme that introduced extensive job assistance and wage subsidies to employers; it was piloted in certain areas of the country and then extended to others. Exploiting variation in age-eligibility criteria and geographical area, the authors find that the programme significantly increased employment among young people aged 18–24.

Hämäläinen et al. (2014) examine the YG programme introduced in Finland in 2005 and consisting of early interventions, monitoring, and tailored job-search plans to guarantee activation measures for unemployed youth. The authors find that the programme moderately increased unsubsidised employment among young people aged 23–24. The reduction in the unemployment rate was otherwise negligible. Furthermore, estimates based on the level of education show that the programme did not improve the labour market prospects of unskilled young people.

Eichhorst et al. (2015) assess the empirical evidence on the effectiveness of vocational education and training programmes in industrialised countries and conclude that training in the form of apprenticeships combined with institutional learning tends to be more effective than school-based training programmes. In the same line, Zimmermann et al. (2013) highlight the advantages of linking school-based training with on-the-job programmes to improve training outcomes for youth in developed and developing countries. In addition, the authors discuss feasible options for implementing vocational training under different economic and institutional conditions, stressing that it is extremely important to engage different institutions to extend vocational training to the informal sector in countries where the latter absorbs a large portion of youth and disadvantaged labour (e.g. NGOs and voluntary organisations).

Evidence on similar programmes in *transition economies* is primarily qualitative.^10^ ALMPs in Latvia have also been analysed by the OECD within the context of the Inclusive Employment Strategy 2015–2020, which was put in place to tackle the large increase in unemployment rates across OECD countries following the global financial crisis. The OECD (2019) report evaluates the impact of activation policies for the unemployed, including formal and non-formal training measures, employment subsidies, and a programme promoting regional mobility, with no specific focus on vocational training implemented under the YG umbrella. Using a dynamic selection-on-observables methodology, it shows that training measures have a positive impact—although the voucher system may compound lock-in effects—while subsided employment was particularly favourable to the long-term unemployed, older people, and young individuals.

Given the more encouraging findings on the effectiveness of training programmes in developing countries,^11^ it is worth stressing a major difference between such programmes and most programmes implemented in developed countries in the 2000s (including the YG): the former are designed to be ‘demand-driven’. That is, training programmes are held in two phases: in the first phase, participants attend a training course in the classroom and are trained for a specific occupation, while the second phase consists of internships in the private sector. The rationale behind ‘demand-driven training’ is twofold. Having certain skills may not be enough for an unemployed person to find a job as their skills must match demand in the labour market.^12^ Thus, it is important that the training provides skills that are in demand by firms. The combination of these two phases is designed to make it easier for participants to enter employment.^13^

To conclude, the recent literature on developing and developed countries tends to agree that dual programmes that link school-based training with (possibly subsidised) on-the-job training are most effective for improving youth employment prospects.

### Evidence on voucher systems

Training programmes can be offered via mandatory assignment by caseworkers or vouchers. In the first case, the caseworker decides which training the unemployed should attend and can impose sanctions in case of no attendance. In the second case, voucher recipients can choose among a set of eligible training providers and courses and are free to not redeem the voucher without fear of sanctions. Hence, the voucher increases recipients’ responsibility and motivation for participating in the training. Moreover, it may enhance competition between training providers (Strittmatter 2016).

The implications of voucher systems are particularly accurately examined in Germany, as several studies have analysed a large-scale reform enacted in 2003 by the German Federal Employment Agency that replaced the mandatory allocation of vocational training programmes with the voucher system (see Doerr et al. (2017), Doerr and Strittmatter (2017), Huber et al. (2018)).

Overall, the empirical evidence shows that compared to mandatory assignment systems, voucher systems may prolong unemployment for voucher awardees, who reduce their job search efforts while looking for a suitable programme, creating a sort of lock-in effect (Strittmatter 2016). Among them, non-redeemers can be trapped in unemployment due to unemployment state dependency. This may lead to efficiency losses (Doerr and Strittmatter 2017; Huber et al. 2018). Similarly, voucher redeemers, i.e. awardees who participate in training, reduce their job search efforts not only while choosing a training programme but also in the participation phase as well. However, in the longer term, training may improve employment opportunities for participants (Strittmatter 2016). Similarly, Doerr et al. (2017) find that being awarded a training voucher has strong negative lock-in effects on both employment and earnings in the short run but can lead to significant positive employment effects after four years from voucher assignment. For the Netherlands, Hidalgo et al. (2014) find that when training vouchers are assigned to low-skilled workers, vouchers or training participation has no significant impact on wages in the short run for low-skilled workers, although they may have a significant and substantial impact on future training plans.

To sum up, voucher systems may lead to efficiency losses in the short term due to a lock-in effect, which may increase unemployment duration for targeted individuals. These losses may be particularly severe for non-redeemers, i.e. awardees who decide not to use the voucher. However, these losses may be compensated by better employment opportunities in the longer term for voucher recipients who decided to attend training.

## The Youth Guarantee and the VT programme under evaluation

The YG is a recent EU initiative aimed at helping unemployed youths enter employment or re-enter the education system. Under the YG umbrella, EU member states commit to implementing measures that ensure unemployed youths receive suitable job offers, education plans, apprenticeships, or traineeships within *4 months* of leaving school or becoming unemployed. The Latvian YG plan is financed through the YEI funds, the European Social Fund (ESF), and the Latvian State Budget, and is managed by the SEA.

In Latvia, the YG consists of a series of ALMPs targeted at young people who meet the following eligibility criteria: (i) registered as unemployed at the SEA; (ii) aged between 15 and 29.

**Fig. 1:**
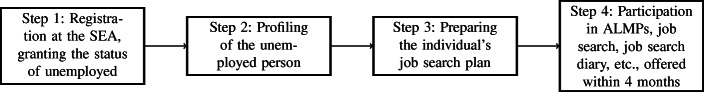
Procedure to enrol in the YG programme. This figure shows the steps for participating in any programme funded by the YG scheme in Latvia

Figure 1 describes enrolment in the Latvian YG programme. Registration at the SEA is the first step, in which an individual is granted unemployment status. Afterwards, unemployed youth undergo a so-called profiling phase during which caseworkers assess their competencies and assist them in their job search, providing tailored-made career guidance (step 2). Within 4 months of registration (steps 3 and 4), unemployed young people should be offered one of the following options, depending on their needs: a satisfying employment opportunity, the opportunity to continue their education, or the opportunity to participate in an ALMP (e.g. an apprenticeship, vocational training).

The programme we consider here is a VT programme offering different types of courses (typically in the classroom) designed to help young unemployed individuals find a job.^14^ It started in January 2014 and was expected to continue until 2020, depending on financial resources. The primary target were youths (i) whose vocational qualifications or professional experience was not in demand on the labour market, leading to difficulties in finding a suitable job; (ii) who had lost their vocational skills; (iii) who had not acquired any vocational qualification at the date of registration at the SEA.^15^ From these criteria, it is already clear that the programme was primarily targeted at the least-employable youths.

If the eligibility criteria for enrolling in the VT programme were fulfilled, the young person received a voucher and could choose a course out of 75 available ones.^16^ The value of a voucher did not exceed 540 euros for programmes of 480 h, 720 euros for programmes of 640 h, or 1100 euros for programmes of 960–1280 h. During the training, each participant received 100 euros per month, plus the reimbursement of travel costs if the course was not offered in their municipality of residence.

Within 10 working days of receiving the voucher offer, the candidate would select the training provider,^17^ which had to check whether the candidate was suitable for participation in the programme. At this point, the SEA officer prepared an agreement between the applicant and the training provider. The latter had to fill out the voucher, sign it, and give it to the applicant, who brought it back to the SEA officer. The contract specified, among other things, the terms and the time of the training, the mutual duties and rights during the training, and the provisions for interruptions and termination of the course. It also specified the organisation of the final examinations. The training had to start within one month of signing the voucher.

To sum up, the programme characteristics may lead to several selection issues that have to be tackled to estimate the causal effect of participating in the VT. First, all individuals in our sample (unemployed youths registered at the SEA) received counselling and job-search assistance provided by the SEA caseworkers. Second, only some of them were offered the VT programme, depending on both their characteristics (their eligibility profile) and the caseworkers’ personal considerations. Finally, young unemployed individuals could freely decide whether to enrol in the training (by redeeming the voucher) or not (*self-selection*). This setting makes it hard to compare participants with non-participants since the latter include both youths who did not receive the VT offer (with different characteristics from participants) and youths who received the VT offer but decided not to enrol (e.g. with similar observable characteristics but different motivation). We seek to address selection as follows. First, we control for individuals’ educational attainment and past employment outcomes to identify the eligible candidates most likely to receive the VT offer based on the conditions described above. Second, we tackle selection issues related to the fact that caseworkers made personal considerations (e.g. caseworkers may have selected the most motivated candidates among those eligible) and that VT is voluntary—that is, individuals self-selected into the programme—in an FRDD framework using individual age-priority rule (see Section 5). In the presence of heterogeneous effects, the FRDD methodology enables us to estimate the causal effect on compliers, i.e. those who participate in the VT thanks to the priority they are given.

### Description of the type of training offered

We now provide some descriptive evidence on the training courses offered, the required skills, and programme lengths. This evidence is useful for the interpretation of the main results in our analysis.

Most courses had a duration of 1–4 months (for more details, see Fig. 5 in Appendix A). Each training course included 8 h of classroom activity per day and did not include an internship. Unfortunately, we do not have detailed information on the teaching evaluations submitted by the participants, as these were collected by the training centres.^18^

The 898 participants we observe in the population are enrolled in 31 different VT courses. We map the VT courses into occupation categories (as if each training course would lead to a certain occupation) using the International Standard Classification of Occupations 2008 (ISCO-08) designed by the International Labour Organisation (ILO). In Table 1, we show the recurrence, the cumulative distribution, the qualification associated with each ISCO category, and the average programme duration.^19^

**Table 1:** Distribution of participants in the training programmes using the ISCO-08 classification

Qualification categories	Freq.	Percent	ISCO skill level	VT duration (months)
Professionals	125	13.92	4	1.5
Technicians and Associate Professionals	209	23.27	3	3.8
Clerical Support Workers	96	10.69	2	2.7
Services and Sales Workers	230	25.61	2	2.0
Craft and Related Trades Workers	213	23.72	2	3.0
Plant and Machine Operators	13	1.45	2	1.3
Elementary Occupations	12	1.34	1	2.8
Total	898	100.00		

This table shows the total number of participants according to the ISCO classification of the main occupation related to the training courses (898 in total)

The courses with the highest numbers of participants are those falling into the following ISCO groups: ‘Services and Sales Workers’ (skill level 2), ‘Technicians and Associate Professionals’ (skill level 3), and ‘Craft and Related Trades Workers’ (skill level 2).^20^ Most youths participated in courses that require a low skill level (mainly skill level 2); 23% of them selected courses with a more technical content, ‘Technicians and Associate Professionals’, and 13% qualified for the category of ‘Professionals’ (skill level 1). Regarding the duration of the training courses, those classified in the categories ‘Technicians and Associate Professionals’ and ‘Craft and Related Trades Workers’ lasted longer, on average—respectively, 3.8 months and 3 months—perhaps due to the higher technical content compared to other courses.

The training courses were held at training centres that are distributed all over the country. Centres are mostly concentrated in the capital city of Riga and in the eastern part of Latvia, which is the least-developed region (Hazans 2005).

It is worth discussing whether these VT courses are attractive and for whom. We notice that there is a concentration of course provision in the least-developed areas in Latvia. As previously mentioned, each participant received 100 euros per month plus the reimbursement of travel costs if the course was delivered in a centre located outside the municipality of residence. This is not an insignificant amount according to Latvian standards and may have attracted individuals just interested in receiving the training allowance. In Latvia, the unemployed can claim unemployment benefits if they had been working for at least 12 months before becoming unemployed. For instance, if a person has worked from 1 to 9 years, the amount of the benefit equals 50% of the average contribution wage. Based on the data received by the Latvian tax authorities, the average gross monthly income for the years we consider was approximately 400 euros, which means that they would likely receive 200 euros (gross) per month, but only if they have worked for at least 1 year. Thus, the programme’s subsidy per se may provide good incentives for participation for the very young who do not have any work experience and for those residing in the least-developed regions of Latvia.

## Data, sample selection, and descriptive statistics

In this study, we use individual administrative records provided by the Latvian Ministry of Finance after merging the data from the SEA with data from the SRS (i.e. the Latvian tax authority). The SEA gathers data on individuals who are registered as unemployed and reports the following information: gender, date of birth, residence, nationality, the highest level of education attained, and exact date of registration, i.e. the starting date of the period of unemployment. In addition, for all VT participants the database contains information on the start and end dates of the training, the type of course attended, and whether the participants completed the course or not. It is also possible to observe whether the individual ever participated in another YG programme before taking part in the VT.

To construct our outcomes of interest, we use administrative data from the SRS, which provides information on the labour market conditions of each individual on specific dates.^21^ This allows us to define an indicator of formal employment at specific dates. For individuals who are formally registered as employed in the SRS database, it is also possible to observe information on their labour income and the firm’s size and industry. This information was extracted for January 2012, June 2012, December 2012, June 2013, December 2013, June 2014, December 2014, June 2015, December 2015, June 2016, December 2016, and June 2017.^22^ As the intervention began in January 2014, data collected between January 2012 and December 2013 are useful to construct pre-intervention measures of individual labour market careers (i.e. employment status, monthly income in euros, hours worked, and social contributions). Data collected in June 2016, December 2016, and June 2017 are useful to construct outcome variables on the labour market performance of individuals 1 to 3 years after the VT programme. The SEA database provides access to all individuals listed as unemployed from June 2013 to December 2015. Our starting sample consists of 40,442 individuals and includes all participants in the VT programme under study (treated group) and all registered unemployed individuals who were eligible for the YG but did not participate either in the VT programme under study or in other training programmes managed by the SEA (control group).

Given that youths could enrol in the programme at any time starting from January 1, 2014 (provided that they met the eligibility criteria described in Section 3), we have to impose some selection criteria. First, we select all unemployed individuals who, on the registration date, were between 15 and 29 years of age (dropping 3424 individuals). Second, we restrict the sample to those registering at the SEA from January 1, 2014, to December 31, 2014 (dropping 25,373 individuals).^23^ This restriction is imposed to prevent potentially dynamic sample selectivity issues. Indeed, including the long-term unemployed in the sample, e.g. those registered in 2012 or 2013, may introduce further unobserved heterogeneity in the analysis. For instance, those who first registered at the SEA in 2012 and remained unemployed in 2014 could be particularly disadvantaged (i.e. negatively selected) due to a lack of skills or low effort put into searching for a job. By selecting those registered at the SEA in 2014, we seek to limit these potential concerns by focusing on short-term unemployed youths. These unemployed individuals were eligible for the YG package and received job search assistance after registration at the SEA. Third, for each treated individual, we set a timeframe of 1 year from the date of registration to assess his or her participation in the training programme. Therefore, we restrict the participation period to the first 12 months after registration, to ensure homogeneity of the treatment (dropping 40 treated individuals) and a sufficient timespan to observe their post-training outcomes.^24^ After imposing these selection criteria, we are left with a final sample of 11,603 individuals, with 898 participants (treated group) and 10,705 non-participants (control group). This sample will be further reduced in the FRDD analysis, in which we impose some age bandwidths.

Note that the treatment status is defined as a binary indicator that equals 1 if the individual participated in the VT programme *within 1 year of their registration date* and 0 otherwise. Hence, someone who registered at the SEA on December 31, 2014, and started the VT by the end of December 2015 is included in the treated group.^25^

Finally, we match the SEA data on both the treated and the control group with information on tax records from the SRS register, which allows us to observe individual outcomes at selected dates: June 2016, December 2016, and June 2017. Since participants started the VT at different points in time, this makes it difficult with the data at hand to create symmetric windows for each participant and observe the outcomes exactly 1 year later, 2 years later, and so on. This and other data limitations are discussed in Appendix A.

**Fig. 2:**
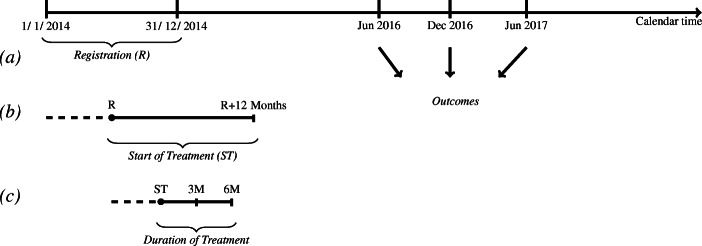
Research design. **a** The estimation sample includes individuals who registered (R) as unemployed at the SEA between January and December 2014; **b** treated individuals are those who started a vocational training programme (ST) within 12 months (M) of registration at the SEA, and control individuals are those who did not participate in any training (neither in that evaluated nor in other types of ALMPs); **c** the duration of the training (treatment) varies between 1 and 6 months

The timeline and the research design are shown in Fig. 2. In line with our selection rules, registration at the SEA takes place between January and December 2014 (a). The treatment starts within 12 months of the date of registration (b) and lasts up to 6 months (c). For example, if one registers on January 1, 2014, and starts the training on the same day, he/she finishes the training by the end of June 2014. For this person, the outcome variables will be measured 2 years after the training (on June 30, 2016) or even later. Conversely, if one registers on January 31, 2014, and starts the training on January 31, 2015 (the latest possible date), he/she completes the training by the end of June 2016. Nevertheless, in our sample, no one started the VT in December 2015. Participation began on September 25, 2015 (at the latest). Thus, at the other extreme, defining the outcome variables on June 30, 2016, means considering short-term effects of at least 6 months after the end of the training. This has implications for our empirical analysis. Given that the outcomes are measured at fixed dates for all individuals, our estimated effects are a weighted average of short-, medium-, and long-term effects (i.e. less than 1 year, between 1 and 2 years, and 2+ years, respectively), with weights depending on the day at which the training starts and the date when outcomes are measured.

Table 2 reports descriptive statistics for the variables used in the analysis, for both the treated and control groups. As shown from the results of the *t*-tests on the difference in the means, the treated and control groups are balanced in terms of nationality, since the proportion of non-Latvian nationals is not statistically different between the groups. In terms of gender, the proportion of women is higher in the treated group than in the control group. The two groups also differ in terms of the level of education and area of residence. First, those in the treated group are, on average, less educated: the proportion of unemployed people with primary education or lower and general secondary education is higher in the treated group than in the control group, whereas the proportion with professional secondary or higher education is higher in the control group. Second, the proportion of individuals living in the capital or in other cities is higher in the control group than in the treated group, with more people in the treated group living in national development centres, regional centres, or rural areas. Finally, the average income and the number of years with positive income in the pre-treatment period (before 2014), which serve as proxies of labour market experience, are higher in the control group than those in the treated group. All in all, these statistics suggest that consistent with the eligibility rules, individuals participating in the VT are less employable in terms of observable characteristics (past work experience, education, etc.) and perhaps also in terms of unobservable characteristics (motivation, job search effort, etc.). This is also in line with VT courses being primarily offered in economically peripheral areas (see Section 3.1).

**Table 2:** Descriptive statistics

	(A)		(B)			
	Controls		Treated		*t*-test	
Variable	Mean	Sd.	Mean	Sd.	Diff.	*t*-stat
Outcome variables						
Employed June 2016	0.452	0.498	0.413	0.493	0.0388*	(2.25)
Employed December 2016	0.420	0.494	0.398	0.490	0.0222	(1.29)
Employed June 2017	0.434	0.496	0.428	0.495	0.00657	(0.38)
Income June 2016	303.621	462.255	204.864	307.492	98.76***	(6.29)
Income December 2016	307.948	513.522	219.927	336.546	88.02***	(5.05)
Income June 2017	326.470	515.588	254.501	367.991	71.97***	(4.10)
Control variables						
Female	0.486	0.500	0.569	0.495	− 0.0834***	(− 4.81)
Foreign nationality	0.374	0.484	0.352	0.478	0.0225	(1.34)
Primary or lower	0.314	0.464	0.392	0.488	− 0.0776***	(− 4.79)
General secondary	0.291	0.454	0.366	0.482	− 0.0749***	(− 4.72)
Professional secondary	0.229	0.420	0.175	0.380	0.0545***	(3.76)
University	0.165	0.371	0.067	0.250	0.0981***	(7.77)
Rural area	0.545	0.498	0.647	0.478	− 0.102***	(− 5.91)
Region: Kurzeme	0.176	0.381	0.194	0.395	− 0.0181	(− 1.37)
Region: Latgale	0.175	0.380	0.324	0.468	− 0.149***	(− 11.03)
Region: Pieriga	0.168	0.374	0.116	0.320	0.0521***	(4.05)
Region: Riga	0.234	0.423	0.109	0.312	0.125***	(8.63)
Region: Vidzeme	0.105	0.306	0.112	0.316	− 0.00785	(− 0.74)
Region: Zemgale	0.143	0.350	0.145	0.352	− 0.00203	(− 0.17)
Average income before 2014	281.419	350.715	180.947	233.529	100.5***	(8.43)
*#* yrs with income> 0 before T	1.903	1.793	1.355	1.621	0.548***	(8.85)
Fraction yrs with income> 0 before T	0.381	0.359	0.271	0.324	0.110***	(8.85)
Observations					11603	

Significance: * *p* < 0.1; ** *p* < 0.05; *** *p* < 0.01. This table shows descriptive statistics (the mean, standard deviation (sd), and sample size) for all dependent and independent variables separately for individuals participating and not participating in VT. In the last two columns, we report the test for the difference in means between the control and the treated. The sample includes all individuals aged 15–29, while in the econometric analysis we apply specific age bandwidths. The sample size for the treated group is 898 observations whereas for the control group is 10,705

## Empirical strategy

Estimating the causal effect of the VT programme on labour market outcomes would be easy if participants were randomly assigned to the programme. This is not the case in our setting as participation was voluntary. Youths were screened by caseworkers based on their vocational qualifications and, after receiving the VT offer, could choose whether to participate or not.

To overcome this identification issue, we leverage a specific feature of the programme, namely the fact that the SEA gave a higher priority for participation in the VT programme to unemployed people under the age of 25, even though the YG targets all individuals in the age range of 15–29.

This feature of the programme makes it ideal for applying an FRDD. In our case, the priority rule and the fact that participation ultimately remained voluntary make the design fuzzy. That is, being subject to the priority rule and being awarded the voucher increased the probability of participating in the programme but did not exactly determine programme participation (as would occur in a sharp RDD).

The idea behind this identification strategy is that, conditional on the observable variables, individuals under the age of 25 were more likely to participate in the VT programme since they had priority. As individuals have no control over their age, around the cut-off age of 25 allocation to the VT programme was ‘as good as randomly assigned’. This means that, on average, treated and control units around the age of 25 should have similar observable and unobservable characteristics but a different probability of receiving VT. This relies on the assumption of ‘no manipulation’, meaning that controls and treated units around the age of 25 could not completely determine their position with respect to the threshold. If this was not the case, individuals with a particular interest in participating may disproportionately lie on one side of the threshold, i.e. by registering at the SEA with the specific purpose of participating in VT programmes. The comparison across the threshold would then be biased because individuals on each side of the threshold would be different in terms of unobservables.

In our analysis, age is the individual attribute (or the running variable) that determines the probability of participating in the VT programme, with the age of 25 representing the threshold—or cut-off point—below which participation in the programme increases due to the priority rule. Since we do not know the exact date on which the profiling phase took place, we assume that for each individual in the sample the eligibility conditions for participation in the VT programme were assessed on the day of registration at the SEA. Hence, we measure age on the date of registration at the SEA as a continuous variable.^26^ According to the priority rule, an individual who was older than 25 on the day she registered at the SEA had a lower probability of participating in the programme. In contrast, an individual younger than 25 on the day she registered at the SEA had a higher probability of participating in the programme, thanks to the priority rule. Since participation was voluntary, the probability of participation for those younger than 25 years of age is strictly less than 1.

The running variable is centred at the cut-off point; hence, it is equal to 0 for someone who registered at the SEA on the day of her 25th birthday and takes a negative (positive) value for those who were younger (older) than 25 at the time of the registration. As such, the running variable represents the difference between the age at the time of registration and the cut-off value: larger negative values correspond to individuals exposed to the priority rule for a longer period. Note that since the running variable is measured for each individual on the starting date of the unemployment spell (i.e. the date of registration at the SEA, which is assumed to coincide with the date on which profiling takes place), the probability of participating in the programme for each individual in the sample discretely jumps at the cut-off point.

The FRDD is implemented using a two-stage least squares (2SLS) estimator. In our setting, the discontinuity in the probability of participating in the programme, given by the age-specific rules, can be used as an exclusion instrument for participation status (Angrist and Pischke 2009).

Our equation of interest is:

1 $$ Y_{i} =\beta_{1} T_{i} + f(\tilde{x_{i}}) + \beta^{\top} \mathbf{W}_{i} + \epsilon_{i},  $$

where *Y*_*i*_ is the employment status or monthly wage of individual *i* at a certain point in time after the training was completed (June 2016, December 2016, and June 2017). *T*_*i*_ is the treatment variable, which takes a value equal to 1 if the individual registered at the SEA as unemployed in the period of January–December 2014 and started the VT programme within 1 year of registration. $\tilde {x_{i}}$ is the running variable, i.e. age measured on the date of registration at the SEA and centred at the cut-off point. *f*(⋅) is a polynomial in the running variable, which represents the relationship between the running variable and the outcome. **W**_*i*_ is a vector of individual covariates such as gender, nationality, level of education, residence area, number of years worked, and average income in the pre-treatment period, including an intercept term. *𝜖*_*i*_ is an individual-specific error term. The exclusion instrument used in the 2SLS is a dichotomous indicator for an individual being younger than 25, i.e. the programme priority rule *Z* = **1**(*x* < 25).

The corresponding first-stage equation reads as follows:

2 $$ T_{i} =\gamma_{1} Z_{i} + \gamma_{2} f(\tilde{x_{i}}) + \gamma^{\top} \mathbf{W} + \eta_{i}, $$

where *Z*_*i*_ is the instrument for *T*_*i*_ and *η*_*i*_ is an individual error term. In the analysis, we specify the polynomial in the running variable as quadratic.^27^

To check the sensitivity of the estimates, we estimate these models on increasingly narrower bandwidths defined in terms of the following age groups: 21–29, 22–28, and 23–27.

### Testing the validity of the identifying assumptions

The validity of the FRDD rests on the local randomisation of the treatment status, that is, the fact that individuals are not able to precisely control the running variable. In our case, the running variable is age measured at registration at the SEA, using the exact date of birth.

The underlying identification assumption is that individuals just below and just above the threshold of 25 years are comparable except for their exposure to the priority rule, which only applies to those below the cut-off. Such an assumption may be violated if individuals can anticipate participation in the intervention, that is, when many people below the age of 25 suddenly start registering at the SEA offices (after January 2014) specifically because they want to participate in the VT programme. In this scenario, we would observe a peak in SEA registration by people just under the age of 25. Furthermore, anticipation would bias our results if the anticipatory behaviour was more pronounced in certain selected groups, e.g. the most motivated youngsters.

To make this more explicit, assume that the priority rule was well known to the target population. Assume that the most motivated youths below 25 years of age decide to register at the SEA in 2014 to participate in the training programme. Moreover, assume that the registration rate among youths above 25 remains unaffected since they are aware of having lower chances of participating in the training programme. In this setting, by comparing registered unemployed youths below the age of 25 with those above 25 years of age, we would be de facto comparing youths who are also different in terms of unobserved characteristics (e.g. motivation). This would violate the identifying assumption underlying the FRDD, i.e. that individuals just below and just above the threshold are similar in all respects except for the treatment assignment.

**Fig. 3:**
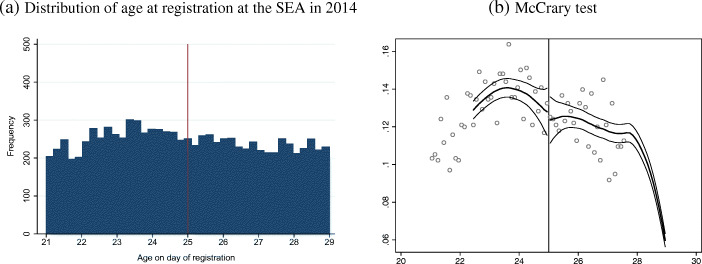
Testing the no-manipulation assumption. In both panels, the x-axis represents the age of registration at the SEA. This figure shows two results. In panel **a**, we show the distribution of the running variable (age on the day of registration) in 2014, and we do not observe any jump around the cut-off. In panel **b**, we report the result of the McCrary test, which shows that the density of the running variable is continuous

In line with what is commonly done in FRDD and RDD studies, to rule out the presence of manipulation we provide two main pieces of evidence. First, we demonstrate that the distribution of the running variable is continuous around the cut-off. Figure 3a clearly shows that this condition is fulfilled. As a second check, we run a formal test as in McCrary (2008), which allows checking the continuity of the density of the running variable around the threshold. The logic behind this test is that if individuals had precise control over the assignment process, we would expect the density of the running variable to exhibit a jump at the cut-off point, being higher to the left of the cut-off (if the treatment is assigned to individuals with smaller values compared to the cut-off and everybody is willing to receive the treatment). Conversely, if individuals have imprecise control over the assignment, the density of the running variable should be continuous around the cut-off. Figure 3b shows that there is no discontinuity in age at the cut-off point, which is evidence in favour of the no-manipulation hypothesis.

Since the absence of manipulation is crucial for our identification strategy, in Appendix A we provide additional evidence to rule out this possibility. First, we want to be sure that the YG programme did not change the incentives for registering at the SEA. Hence, we compare the age distribution at the date of registration at the SEA in 2014 (when the YG started) and in 2013, i.e. before the start of the YG. Figure 7 suggests that the age distribution for registering in 2013 (Fig. 7a) is very similar to the one observed for 2014 (Fig. 7b). In particular, note that in Fig. 7a the vertical red line at 25 years of age indicates the ‘placebo’ priority rule for participating in the training programme when the latter was not yet put in place. Therefore, in 2013, there is no reason to expect a peak in the distribution of age at registration at age 25. The two figures are very similar.

Second, in Fig. 8 we show the number of individuals aged 15–29 registered as unemployed at the SEA by date of entry (from January 2013 until December 2014). The vertical line on January 1, 2014, defines the introduction of the YG. Registration at the SEA is not uniformly distributed over time, but follows a pattern. The number of registrations increases in the second semester of 2013, reaches a peak in January 2014, and then decreases in the first semester of 2014. This does not invalidate our FRDD analysis, however.

Finally, we provide further informal tests showing that there is no discontinuity in other individual characteristics before the treatment takes place (see Fig. 9). This is also a way to show that individuals on either side of the cut-off are balanced in terms of covariates, and so they are comparable. In Fig. 9, we show the distribution of characteristics such as level of education, average income calculated over five distinct points in time before participation in the training programme (between January 2012 and December 2013), and area of residence (dummy for residing in the capital city, Riga). In all of these cases, individuals have similar characteristics around the cut-off. This suggests that individuals on the right and on the left of the threshold are comparable in terms of observable characteristics. Moreover, this also allows us to rule out the possibility that individuals with given characteristics decided to register at the SEA just before turning 25 in order to benefit from the programme.

## Results

In this section, we discuss the main results of our FRDD strategy, potential heterogenous effects, and additional robustness checks on the main model.

### Baseline results

**Fig. 4:**
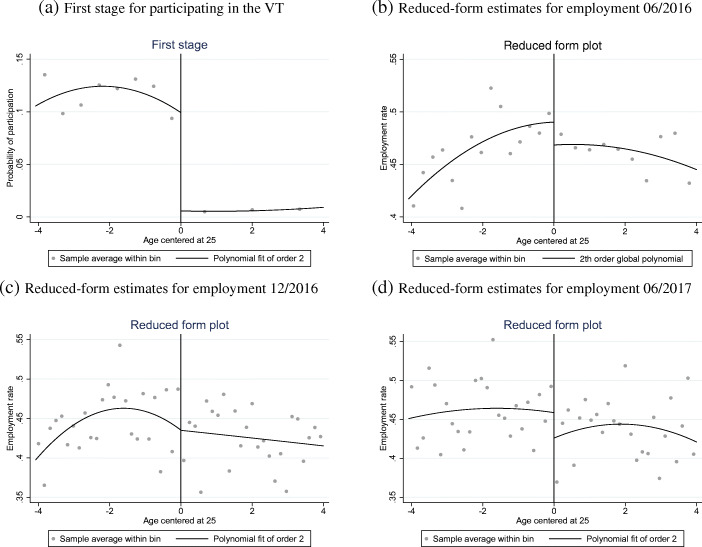
FRDD: first-stage and reduced-form regressions. **a** plots the results from the first stage, the probability of participating in the VT programme as a function of age at registration at the SEA. Age is continuous and measured in days. We use a quadratic specification in age and do not condition on other covariates such as gender, nationality, and region of residence. **b**, **c**, and **d** plot the results from the three reduced-form specifications, namely the probability of being employed at different dates—June 2016 (top right), December 2016 (bottom left), and June 2017 (bottom right)—as a function of age at SEA registration, normalised at the cut-off point

**Fig. 5:**
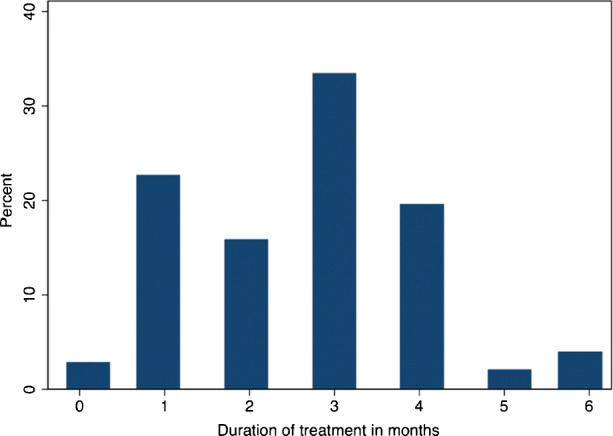
Duration of training in months

**Fig. 7:**
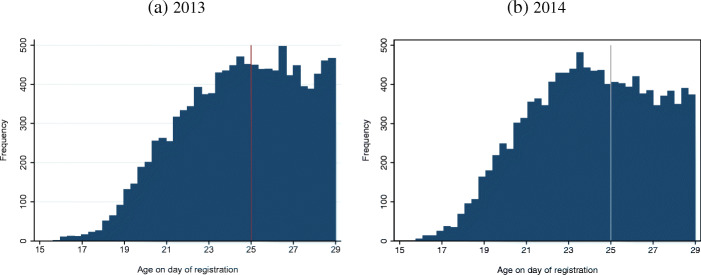
Distribution of age at registration at the SEA in 2013 and 2014. This figure shows the distribution of age at registration at the SEA in 2013, before the start of the YG, and in 2014 when the YG was in place

**Fig. 8:**
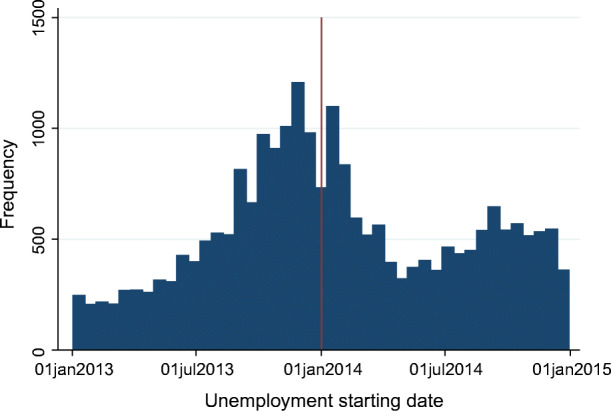
Unemployed (age 15–29) registered at the SEA in 2013–2014. This figure shows the number of individuals aged 15–29 registered as unemployed at the SEA between January 2013 and December 2014

**Fig. 9:**
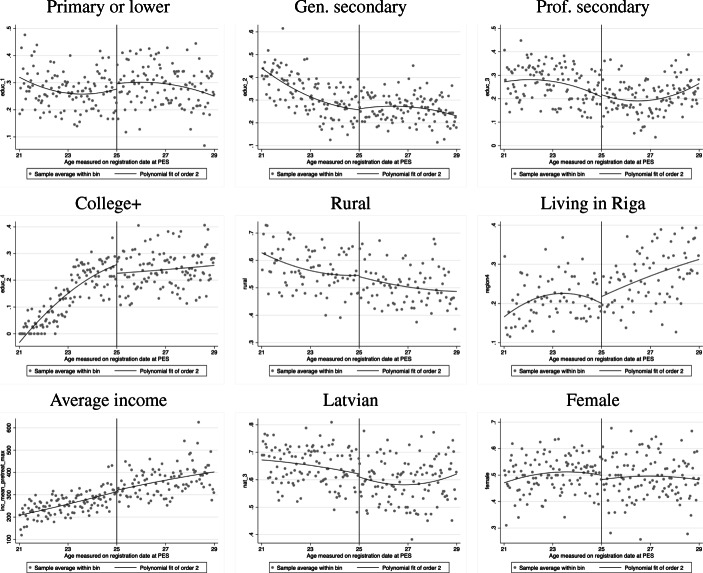
Testing for continuity of control variables. This figure reports the results of informal tests. It shows that there is no discontinuity around the cut-off for all of the other covariates used in our model, namely, level of education (primary or lower, general secondary, professional secondary, college or more), indicators for living in a rural area or in the capital city, average income, an indicator for being a citizen of Latvia, and gender (indicator for female)

We first show the results of the first-stage (i.e. the effect of the priority rule on participation in the VT programme) and the reduced-form estimations (i.e. the effect of the priority rule on the outcome variables). Figure 4a plots the probability of participating in the VT programme as a function of age at registration at the SEA, where age is measured in years, starting from age in days. We use a quadratic specification in age and do not condition on the covariates. We notice a clear jump in the probability of participating in the VT at the cut-off (age 25), due to the priority rule.

Figures 4b, 4c, and 4d show the probability of being employed at different dates—June 2016 (top right), December 2016 (bottom left), and June 2017 (bottom right)—as a function of age at SEA registration, normalised at the cut-off point. In contrast to Fig. 4a, we do not observe a clear jump at the cut-off.

Table 3 shows the results of the first-stage equation for the quadratic polynomial specification using different age bandwidths, namely 21–29, 22–28, and 23–27. Being subject to the priority rule (i.e. being younger than 25 years of age) increases the probability of participating in the programme by about 11.1 pp when considering youths between the ages of 21–29, which drops to 8.9 pp when considering those in the 23–27 age group. Note that the larger the bandwidth around the cut-off, the higher the precision of the estimates as we are using more data points to fit our model. Wider age bandwidths imply that the estimates tend to be less reliable (higher bias) due to the fact that we are using data points that are far away from the cut-off. Interestingly, even focusing on individuals with more similar ages (imposing the age bandwidth), VT participants are more likely to be female, less educated, not have professional secondary education, and live in peripheral areas, which we already observed in Table 2.

**Table 3:** First-stage results for the probability of participating in the VT programme

Age bandwidth	21–29		22–28		23–27	
Age < 25	0.111	***	0.105	***	0.089	***
	(0.010)		(0.011)		(0.014)	
Age centred at 25	0.002		− 0.002		− 0.011	*
	(0.002)		(0.003)		(0.006)	
Age centred at 25, squared	0.000		− 0.000		0.005	*
	(0.001)		(0.001)		(0.003)	
Female	0.030	***	0.032	***	0.039	***
	(0.005)		(0.006)		(0.007)	
Foreign nationality	0.002		0.002		0.008	
	(0.006)		(0.006)		(0.008)	
General secondary	0.010		0.007		0.003	
	(0.007)		(0.008)		(0.009)	
Professional secondary	− 0.018	***	− 0.020	**	− 0.016	*
	(0.007)		(0.008)		(0.010)	
University	− 0.027	***	− 0.033	***	− 0.039	***
	(0.008)		(0.009)		(0.010)	
Rural area	0.004		0.005		0.006	
	(0.007)		(0.008)		(0.009)	
Region: Kurzeme	0.026	***	0.027	***	0.026	**
	(0.009)		(0.010)		(0.012)	
Region: Latgale	0.061	***	0.060	***	0.059	***
	(0.009)		(0.010)		(0.012)	
Region: Pieriga	0.001		0.002		0.002	
	(0.010)		(0.011)		(0.013)	
Region: Vidzeme	0.021	**	0.013		0.015	
	(0.011)		(0.012)		(0.015)	
Region: Zemgale	0.021	**	0.021	*	0.021	
	(0.009)		(0.011)		(0.013)	
Fraction of years with positive income	0.003		0.001		0.006	
	(0.008)		(0.010)		(0.012)	
Average income before 2014	− 0.000		0.000		0.000	
	(0.000)		(0.000)		(0.000)	
N. obs.	9623		7491		5141	
*F*-stat	135.29		91.52		43.76	
*N*. treated	616		486		338	
*N*. controls	9007		7005		4803	

Significance: * *p* < 0.1; ** *p* < 0.05; *** *p* < 0.01. This table shows the estimated coefficients from the first-stage regressions, using a quadratic polynomial in age. The columns show the results for different age bandwidths: 21–29, 22–28, 23–27. The excluded categories are male, Latvian national, primary education or lower, urban area, Riga region. Standard errors are in parentheses

In all specifications, we use as outcomes of interest an indicator for being employed in June 2016, December 2016, and July 2017, and one’s gross monthly income expressed in euros^28^ at the same dates.

Tables 4 and 5 are organised into three different panels showing the estimates for outcomes measured on June 2016, December 2016, and June 2017, respectively. It is worth noting again that outcomes at later dates show relatively more medium-term and long-term effects compared to outcomes at earlier dates. For each outcome, we report results from the ordinary least square (OLS) specification, the reduced form, and ‘local IV’ (FRDD) estimates via 2SLS, for different age bandwidths around the cut-off. We use a quadratic specification of the polynomial in the running variable in our baseline estimates.

**Table 4:** Results for the probability of being employed at specific dates

Outcome		Jun. 2016			Dec. 2016			Jul. 2017	
Age	21–29	22–28	23–27	21–29	22–28	23–27	21–29	22–28	23–27
OLS									
Treated	0.001	0.005	0.007	0.018	− 0.002	0.014	0.021	0.018	0.019
	(0.021)	(0.023)	(0.028)	(0.021)	(0.023)	(0.028)	(0.021)	(0.023)	(0.028)
Reduced form									
Age< 25	0.016	0.018	0.009	0.016	− 0.003	0.007	0.013	0.012	0.035
	(0.019)	(0.022)	(0.027)	(0.019)	(0.022)	(0.027)	(0.019)	(0.022)	(0.027)
2SLS									
Treated	0.147	0.175	0.103	0.140	− 0.029	0.078	0.117	0.112	0.386
	(0.176)	(0.213)	(0.305)	(0.174)	(0.211)	(0.302)	(0.174)	(0.211)	(0.308)
*F*-stat	135	91.5	43.8	135	91.5	43.8	135	91.5	43.8
*N*. obs.	9623	7491	5141	9623	7491	5141	9623	7491	5141
*N*. treated	616	486	338	616	486	338	616	486	338
*N*. controls	9007	7005	4803	9007	7005	4803	9007	7005	4803

Significance: * *p* < 0.1; ** *p* < 0.05; ***p < 0.01 This table shows the OLS, reduced form, and 2SLS results, using an indicator for being employed in June 2016, December 2016, and June 2017 as main outcomes. We use a quadratic specification for age. The columns show the results for different age bandwidths: 21–29, 22–28, and 23–27. The control variables are not shown, but we use the same set of covariates as reported in the first-stage regressions. Standard errors are in parentheses

**Table 5:** Results for monthly income at specific dates

Outcome		Jun. 2016			Dec. 2016			Jul. 2017	
Age	21–29	22–28	23–27	21–29	22–28	23–27	21–29	22–28	23–27
OLS									
Treated	− 44.519**	− 55.900***	− 47.769*	− 30.027	− 56.390**	− 44.342	− 21.798	− 37.115	− 27.909
	(18.610)	(21.215)	(25.693)	(20.947)	(23.267)	(27.769)	(21.072)	(23.645)	(28.806)
Reduced form									
Age< 25	10.024	3.321	− 2.584	8.783	− 8.072	5.986	6.560	6.657	12.562
	(17.546)	(20.314)	(24.968)	(19.746)	(22.277)	(26.982)	(19.863)	(22.634)	(27.986)
2SLS									
Treated	90.188	31.523	− 28.878	79.022	− 76.626	66.892	59.026	63.192	140.382
	(158.023)	(192.611)	(278.181)	(177.636)	(211.011)	(301.107)	(178.581)	(214.687)	(312.909)
*F*-stat	135	91.5	43.8	135	91.5	43.8	135	91.5	43.8
*N*. obs.	9623	7491	5141	9623	7491	5141	9623	7491	5141
*N*. treated	616	486	338	616	486	338	616	486	338
*N*. controls	9007	7005	4803	9007	7005	4803	9007	7005	4803

Significance: * *p* < 0.1; ** *p* < 0.05; *** *p* < 0.01. This table shows the OLS, reduced form, and 2SLS results using gross monthly income registered in June 2016, December 2016, and June 2017 as the main outcomes. We use a quadratic specification for age. The columns show the results for different age bandwidths: 21–29, 22–28, and 23–27. The control variables are not shown, but we use the same set of covariates as reported in the first-stage regression. Standard errors are in parentheses

OLS estimates from Table 4 show that participating in the VT programme has a positive but very small and not statistically significant association with the probability of being employed in June 2016, varying in the 0.1–0.7 pp range according to the choice of bandwidth. However, as discussed in the previous section, OLS estimates could be biased as individuals may be chosen by caseworkers on the basis of unobserved characteristics such as motivation or ability. They may still be useful to assess the incidence of selection bias when they are compared with the FRDD estimates, however.

In the reduced form estimates, being younger than 25 (i.e. subject to the priority rule) raises the probability of being employed in June 2016 by 0.9–1.8 pp depending on the bandwidth, although the result is again not statistically significant. Finally, the 2SLS estimates also show no statistically significant effect of participating in the VT programme in terms of employability, with much larger coefficients falling in the 10–18 pp range. The larger magnitude of the 2SLS estimates compared to the reduced form ones is consistent with the relatively low proportion of those who comply with the rule (the probability of participation in VT based on first-stage results is around 10 pp).

We reach the same conclusion when looking at the effect of participation in the VT programme on the probability of being employed in December 2016 or June 2017. Except for one case,^29^ the estimated coefficients from the OLS, reduced form, or 2SLS specifications have similar magnitudes to those observed in June 2016 and are never statistically significant.

We now discuss Table 5, where we report the estimates for gross monthly labour income (in euros) as of June 2016, December 2016, and June 2017 (income is coded as 0 for unemployed individuals). As before, the OLS, reduced form, and 2SLS estimates are generally not statistically significant, with the exception of the OLS estimates for June 2016, which show a negative sign with coefficients ranging from − 45 to − 56 euros. Interestingly, the OLS estimates are always negative, irrespective of the date at which they are measured. This result may reflect the negative selection of the individuals participating in the VT, especially in terms of earnings capacity. This selection bias in the OLS estimates is, however, removed by the FRDD. Indeed, when looking at the 2SLS estimates, we notice that VT increases monthly income at June 2016 by 90 euros among the 21–29 age group, although this is statistically nonsignificant. Compared to the employment outcomes, the coefficient magnitudes are more sensitive to the bandwidth choice. They are usually positive, except for a negative coefficient for the 23–27 bandwidth. A similar pattern is observed when measuring monthly labour income in December 2016 or June 2017.

A general concern with the estimates based on the FRDD is that the magnitude of the 2SLS estimates is greater in absolute value compared to the OLS estimates. This could either be due to a lack of precision (especially in the narrower bandwidths, in which the number of treated individuals is relatively small), to the presence of weak instruments, or to the local nature of the FRDD estimates. However, strong positive effects from the first-stage equation and the lack of statistical significance in the reduced-form regressions seem to exclude weak instruments as the primary reason for the statistically nonsignificant effects.

As is well explained by Abadie (2020), even estimates that are not statistically significant can provide useful information. Several pieces of evidence from our analysis, if jointly read with the extant literature reviewed in Section 2 and the main features of the VT programme under evaluation, pushes us towards a more positive reading of our results than a simple consideration of statistical significance may suggest. First, the programme had many features that might have made it not very effective, according to the findings of past programme evaluations. Specifically, it was mainly based on classroom training, while scholars have generally found that on-the-job training is more effective to help the unemployed find a job; it was not coupled with demand-side measures (e.g. hiring subsidies or employment vouchers, see Orszag and Snower (1999)); it was based on a voucher system, which might have induced further lock-in effects; and the fact that participants were given a subsidy (see Section 3.1) might have attracted low-motivated individuals to the programme, i.e. people participating only to receive the monetary contribution. Aside from this, our sample sizes are admittedly not very large, in particular in terms of the number of treated individuals, and we might lack enough statistical power to estimate precise effects. On the other hand, the estimated 2SLS coefficients are quite often positive. This is worth noting especially in light of the eligibility criteria that caseworkers had to follow when offering VT participation, which was primarily targeted to the least-employable youths. The latter and the fact that our estimates may still suffer from a downward bias if the control variables (e.g. individual education, past incomes) are not able to fully account for initial eligibility selection made by caseworkers make us conclude that even if the VT was not able to create an employment or earnings advantage compared to the control group, it was nonetheless capable of removing the employment and earnings gaps that treated individuals had ex-ante, i.e. at the moment they were offered the training vouchers.

A further consideration to be made is that, from a parametric point of view, estimating an FRDD is equivalent to estimating a ‘local IV’ (i.e. in the vicinity of the cut-off) model. In a heterogeneous-effect setting, this implies that the 2SLS estimator provides local estimates on the subpopulation of the so-called *compliers*, i.e. individuals whose VT participation status is affected by the priority rule. Therefore, it is useful to know as much as possible about the characteristics of this subpopulation.

In the next section, we analyse more closely the characteristics of compliers. Our analysis indeed confirms that compliers are more likely to be drawn from among the least-employable individuals, somehow confirming our positive assessment of the programme’s results.

### Characteristics of the *compliers*

Each instrument helps identify a unique causal parameter. Using an FRDD setting, at best we can identify the ‘Local Average Treatment Effect’ (LATE) for the group of compliers, that is, the subpopulation whose treatment status (participation in the YG or non-participation) is determined by the instrument (the priority rule).

The compliant subpopulation associated with the priority rule instrument is composed of youths who, in absence of the rule, would not have participated in the training programme.

Although the compliers are not observed, we can learn something about their characteristics by exploiting Bayes’ theorem when both the endogenous variable and the instrument are binary.^30^ This can be done based on pre-treatment characteristics (*X*_*i*_), which follow a Bernoulli distribution (binary indicators), in order to answer questions such as the following: Are compliers in the VT programme more likely to be female or reside in rural areas compared to the full sample? For this exercise, we use binary indicators that refer to the pre-treatment period: an indicator for being female, an indicator for having Latvian citizenship, an indicator for living in a rural area, and one for living in the capital city; four indicators for the level of education (i.e. primary education or lower, general secondary, professional secondary, and university); and finally, an indicator for not having any income before participation in the training programme.

Table 6 summarises our results. We report the following values: unconditional mean of the pre-treatment dummy variables (*X*) calculated over the whole sample (column 3), conditional mean for the complier subpopulation (column 4), and the relative likelihood for a complier of having *X* = 1, that is, the ratio between column 4 and column 3 (column 5).

**Table 6:** Characterisation of the compliant population

Exogenous variable (X)	*Pr*(*X* = 1)	*Pr*(*X* = 1|compliers)	$\frac {Pr(X=1 | \text {compliers})}{Pr(X=1)}$
Female	0.492	0.561	1.140
Latvian citizenship	0.627	0.634	1.011
Rural area	0.553	0.626	1.133
Riga	0.224	0.119	0.529
Primary or lower	0.320	0.388	1.213
General secondary	0.297	0.338	1.139
Professional secondary	0.225	0.150	0.668
University	0.157	0.096	0.608
No income before T	0.344	0.414	1.206

Significance: *p < 0.1; **p < 0.05; *** *p* < 0.01. The total number of observations is 11,603. See Angrist and Pischke (2009), pages 261–263 for the details

Compared to the whole sample, compliers are 14% more likely to be female (the ratio is equal to 1.14), 1% more likely to have Latvian citizenship,^31^ 13% more likely to live in rural areas, and 52% less likely to live in Riga. Concerning educational achievement, we find that the compliers are 21% more likely to have primary education or lower and approximately 14% more likely to have a general secondary education. Conversely, they are less likely to have a professional secondary or university education. Finally, we find that compliers are 20% more likely to have had no income in the period of 2012–2013.^32^ All in all, these characteristics confirm that the compliers, on average, seem to have poorer skills and lower employability compared to the whole sample, which is consistent with the eligibility rules and aim of the VT programme.

### Robustness checks

In this section, we discuss a series of robustness checks that we have carried out.

First, to further check the sensitivity of our estimates to dynamic selection issues in the baseline estimates, we run the analysis defining the treatment as participation in the training within a window of 6 months since initial unemployment registration, instead of 12. Results are in line with the main findings; namely, we do not find any significant effect of the programme on employment at any point in time.^33^ Second, we use different specifications for the FRDD, i.e. a linear polynomial. The results do not change.^34^ Third, in the main regressions we include an additional control for the time elapsed between the date of registration at the SEA and the time at which the outcome is measured (June 2016, December 2016, June 2017), and the results are very similar.

An issue with our FRDD results is that the coefficients seem to be imprecisely estimated and sometimes large in magnitude, although the instrument does not appear to be weak. A possible reason is the fact that we are left with few observations around the threshold, especially when restricting the analysis to smaller bandwidths. To tackle this issue, we provide as a robustness check estimates of the effect of VT on employment and monthly income using propensity score matching (PSM) techniques. We use nearest-neighbour matching with 1 and 5 matches within the same age intervals used for FRDD. Average treatment effects on the treated (ATETs) are reported in Tables 7 and 8.

**Table 7:** Propensity score matching: results for the probability of being employed on specific dates

Employment		Jun. 2016			Dec. 2016			Jul. 2017	
Age	21–29	22–28	23–27	21–29	22–28	23–27	21–29	22–28	23–27
PSM – 1 nearest neighbour									
ATET	0.016	0.029	0.033	0.010	0.027	− 0.018	0.006	0.035	− 0.018
	(0.029)	(0.034)	(0.040)	(0.029)	(0.033)	(0.040)	(0.028)	(0.033)	(0.040)
PSM – 5 nearest neighbours									
ATET	0.008	0.032	0.007	0.017	0.014	0.017	0.029	0.031	0.008
	(0.023)	(0.026)	(0.032)	(0.023)	(0.026)	(0.030)	(0.022)	(0.026)	(0.030)

Significance: * *p* < 0.1; ** *p* < 0.05; *** *p* < 0.01 This table shows the ATET (average treatment effect on the treated) estimates using propensity score matching techniques (nearest-neighbour matching), using as outcomes indicators for being employed in June 2016, December 2016, and June 2017. The results are obtained using the teffects Stata package. The columns show the results for outcomes measured on specific dates for each age bandwidth: 21–29, 22–28, and 23–27. The matching variables are the control variables reported in the baseline FRDD model

**Table 8:** Propensity score matching: results for monthly income on specific dates

Incomea		Jun. 2016			Dec. 2016			Jul. 2017	
Age	21–29	22–28	23–27	21–29	22–28	23–27	21–29	22–28	23–27
PSM–1 nearest neighbour									
ATET	− 44.919**	− 21.907	− 27.661	− 39.178*	− 41.147	− 61.803*	− 32.174	− 18.221	− 36.088
	(21.908)	(24.521)	(28.963)	(23.259)	(29.096)	(32.300)	(23.697)	(28.939)	(33.306)
PSM–5 nearest neighbours									
ATET	− 42.776***	− 32.026*	− 60.782***	− 29.232*	− 43.793**	− 56.065**	− 16.288	− 27.611	− 46.666*
	(15.170)	(17.277)	(22.379)	(16.690)	(19.054)	(23.083)	(17.350)	(20.123)	(24.281)

Significance: * *p* < 0.1; ** *p* < 0.05; *** *p* < 0.01 This table shows the ATET (average treatment effect on the treated) estimates using propensity score matching techniques (nearest-neighbour matching), using as outcomes labour incomes in June 2016, December 2016, and June 2017. The results are obtained using the teffects Stata package. The columns show the results for outcomes measured on specific dates for each age bandwidth: 21–29, 22–28, and 23–27. The matching variables are the control variables reported in the baseline FRDD model

As can be seen from Table 7, we still obtain positive but statistically nonsignificant effects on employment. We are aware that PSM is based on different identifying assumptions compared to FRDD, yet we deem PSM estimates informative as they are less local and suffer less from small-sample issues.^35^ The PSM estimates on earnings, in Table 8, provide a different picture. The estimated effects of VT are indeed negative and often statistically significant, especially when more neighbours are used to compute the ATET. Given that the PSM methodology, unlike FRDD, cannot account for selection on unobservables (ability, motivation, etc.), the PSM results suggest that especially individuals with a low earnings potential might have selected into VT.

In Table 9 in Appendix A, we show the PSM results by considering two age groups: 21–24 and 25–29, namely, below and equal to and above 25 years of age. As for employment, the point estimates are generally positive and larger at later dates (consistent with larger ‘lock-in effects’ immediately after programme participation) and for older individuals (25–29), especially when more neighbours are considered, but they are usually statistically nonsignificant. The estimates on incomes tend to be negative in the short run and apparently remain negative also in the medium and long run for younger individuals (21–24), although they become statistically nonsignificant.

Finally, we combine PSM with the FRDD estimates. The idea is to compare VT participants with non-participants with very similar observable characteristics. PSM-FRDD is implemented using inverse probability weighting (i.e. observations are weighted by the probability of treatment). The results are reported in Table 11 in Appendix A. Also in this case, we observe a striking difference between the weighted OLS estimates—which are almost always negative (and statistically significant for income)—and the FRDD estimates that are positive and statistically nonsignificant. By and large, we interpret this evidence as PSM not being able to completely remove selection bias in the estimation. Importantly, the conclusions of our analysis do not change.

## Conclusion

In this paper, we evaluate the causal impact of a vocational training programme on employment outcomes. This training was implemented in Latvia as part of the Youth Guarantee scheme and targets unemployed youths aged 15–29 who registered at the Latvian public employment office. Our analysis is based on rich administrative data provided by the Latvian public employment office and matched with data from the Latvian Tax Agency. To estimate the causal effect of participation in the programme on subsequent employment outcomes, we rely on a fuzzy regression discontinuity design, leveraging a specific eligibility criterion adopted by the Latvian government that gave priority to young people under the age of 25 for the training. In this setting, this priority rule provides presumably exogenous variation for youth participation in the programme, thereby allowing us to estimate its causal effect on labour market outcomes (employment status and income) later in life by netting out confounding effects mainly due to selection into the programme.

Our results show that the priority rule strongly predicts youth training participation. In contrast, the programme yields no statistically significant positive effect either on employment status (dependent employment in the formal sector) or on declared labour income after training.

Our results are broadly consistent with the extant literature on active labour market policies targeted at youths, which often finds little-to-no-effect due to the presence of lock-in effects or to the fact that these measures are not coupled with demand-driven interventions (e.g. tax rebates or firm-provided training). In the specific case of the programme under evaluation, we claim that a combination of factors might have produced this effect, namely, the eligibility criteria for the programme—which targeted the least-employable individuals—lock-in effects produced by the voucher system on top of those induced by programme participation, and the specific type of training involved (i.e. classroom rather than on-the-job training). On the other hand, our assessment of the programme is not completely negative, especially in light of the negative selection characterising the control group. Our study points to the fact that the programme was at least capable of removing some of the employment and earnings barriers that treated individuals suffered from ex ante, allowing them to perform similarly to the control group on the labour market after training.

Our study provides first evidence of the impact of an intervention financed with the Youth Guarantee. Increasing our knowledge of the effectiveness of the 2014–2020 Youth Guarantee funds is key, especially in light of the labour market conditions produced by the recent pandemic. The socio-economic impact of the pandemic on the youth is undisputed. Recent evidence documents labour market disruptions and job losses for young workers during the early months of the COVID-19 recession in the USA (Montenovo et al. 2020; Cortes and Forsythe 2020). For Europe as well, young people are identified as one of the most vulnerable categories of individuals (Foucault and Galasso 2020) and those most exposed to earnings losses (Bell et al. 2020). Given that the COVID-19 crisis is affecting young people disproportionally, the European Commission is fine-tuning its actions to support youth employment. The recently launched 2020 Youth Employment Support aims to modernise vocational education and training to make the transition from school to work smoother and boost apprenticeships. Moreover, it includes a reinforced Youth Guarantee program to offer comprehensive job support across the EU to a broader target group of 15- to 29-year-olds.^36^ Having reached over 24 million young people who registered in Youth Guarantee schemes starting with the first scheme in 2013, the reinforced Youth Guarantee intends to support youths at risk of unemployment and activate the hardest-to-reach through tailored, individualised approaches and guidance. In this context, it becomes more urgent than ever to provide more evaluations of past Youth Guarantee programmes in order to gather evidence on the features of successful training and spend the huge amount of resources that EU is allocating to fighting youth unemployment in an effective way.

## Figures and Tables

**Fig. 6:**
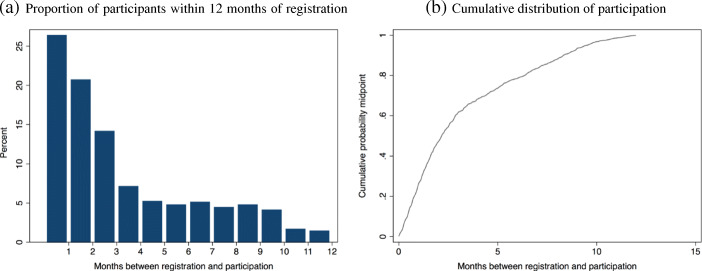
Participation in the programme within 12 months of registration

## Appendix

### 2: Data limitations

Here we discuss some of the limitations of our study, mostly related to data availability. First, with the data at hand, we are not able to construct the sample as a balanced panel—that is, to observe each individual after the same amount of time (6 months, 1 year, etc.) from the date of completing VT. In our setting, individuals can start the training at any time between January 2014 and December 2014, and the data from the tax records allow us to observe the outcomes of the treated and control groups (indicators for being employed and monthly incomes) at 3 points in time: June 2016, December 2016, and June 2017.^37^

Second, as explained in Section 3, training programmes are typically managed through a voucher system: young unemployed individuals receive a voucher that can be spent in one of the country’s vocational education institutions. One concern is the fact that some individuals who receive the voucher may decide not to use it, and with the data at hand we are not able to observe this subgroup. Based on information from the SEA, this amounts to 18% of all voucher recipients, on average.^38^ The reasons for not redeeming the voucher are varied and may include finding a job, health problems, and lack of motivation, but also delays in the process as it may take more than 4 months to start the training. About 10% of total voucher receivers are dropouts, i.e. they receive the voucher and start the training but do not finish it. The reasons behind this may be similar. In any case, if the participant finds a job, she has still the possibility to finish the training while on the job.

**Table 9:** Propensity score matching. Separate estimates for youths below and above age 25

	Age 21–24	Age 25–29	Age 21–24	Age 25–29
Employed – Jun. 2016		Income – Jun. 2016		
PSM – 1 nearest neighbour				
ATET	0.036	− 0.033	− 18.293	− 79.014*
	(0.033)	(0.106)	(22.348)	(47.336)
PSM – 5 nearest neighbours				
ATET	0.010	− 0.088	− 43.370**	− 103.419***
	(0.026)	(0.068)	(17.838)	(35.242)
Employed – Dec. 2016		Income – Dec. 2016		
PSM – 1 nearest neighbour				
ATET	0.029	0.067	− 9.961	27.170
	(0.032)	(0.106)	(24.156)	(59.847)
PSM – 5 nearest neighbours				
ATET	0.007	0.179***	− 30.373	74.532
	(0.027)	(0.007)	(19.546)	(76.972)
Employed – Jun. 2017			Income – Jun. 2017	
PSM – 1 nearest neighbour				
ATET	0.064*	0.000	1.739	18.105
	(0.034)	(0.114)	(26.735)	(86.225)
PSM – 5 nearest neighbours				
ATET	0.025	0.106	− 23.938	− 0.196
	(0.026)	(0.072)	(20.571)	(63.359)

Significance: **p* < 0.1; ** *p* < 0.05; ****p* < 0.01

This table shows the ATET (average treatment effect on the treated) estimates using propensity score matching techniques (nearest-neighbour matching), using labour outcomes (probability of employment, labour incomes) in June 2016, December 2016, and June 2017. The results are obtained using the teffects Stata package. The columns show the results for outcomes measured on specific dates for each age bandwidth: 21–29, 22–28, and 23–27. The matching variables are the control variables reported in the baseline FRDD model

**Table 10:** Percentage of workers aged 15–29 in various sectors for the period of 2012–2014: EU-LFS for Latvia (self-employed) vs Latvian tax records (all workers)

	EU-LFS (self-employed)	Latvian tax data (all workers)
Agriculture	19.17	1.86
Mining and quarrying	0.00	1.32
Manufacturing	3.07	1.05
Electricity	0.00	2.29
Water supply	0.00	28.34
Construction	6.54	1.48
Wholesale	3.67	7.87
Transportation	2.50	0.79
Accommodation	1.66	3.73
Information	9.94	16.76
Financial activities	0.00	2.16
Real estate activities	2.56	5.32
Professional activities	13.50	5.32
Administrative activities	6.22	4.08
Public administration	0.00	1.63
Education	2.48	0.30
Human health	0.72	3.24
Arts	1.67	9.66
Other service activities	28.76	2.80

Author’s calculations. We show the percentage of self-employed workers aged 15–29 out of the total employed across different sectors. The first column uses data from the EU-LFS. The second column uses Latvian tax data. In both cases, we pool the data for the years 2012–2014 and use the NACE job-sector classification (first digit)

Third, since we use information from tax records to check the employment status of both participants and non-participants after the end of the training (2015–2017), we are only able to observe formal employment, disregarding other work arrangements such as self-employment. Furthermore, as in other studies, it is not possible to determine whether individuals continue their

education after the training experience, whether they migrate, or whether they commute to work in neighbouring countries (e.g. Estonia, Lithuania). Disregarding self-employment may bias the impact of the programme downwards if the programme were to boost entrepreneurship and contribute to the creation of new jobs. Unfortunately, tax registers are not integrated in Latvia, so only monthly income for a job spell from formal employment is available in our data. More precisely, the SRS collects tax declarations only for self-employed persons earning an income above a certain threshold (i.e. above 213 euros, according to Eurofound). To check for the presence of any self-employment prevalence among our VT participants and non-participants, we exploit information on the industry in which they worked before the implementation of the YG, namely, before January 2014. We rely on the Statistical Classification of Economic Activities in the European Community (NACE) code, using a one-digit classification. We then compare the distribution of participants and non-participants in our data across different sectors, with the distribution of the self-employed across sectors obtained using data on Latvia from the European Union Labour Force Survey (EU-LFS).^39^ Since the EU-LFS provides information on individuals’ ages using 5-year bands (0–4, 5–9, 10–14, 15–19, and so on), we look at the 15–29 age group (the policy’s target group).

Based on the EU-LFS data for the period of 2012–2014 (Table 10), the three sectors with the highest share of self-employed individuals are ‘Agriculture’ (19.2%), ‘Other service activities’ (28.8%), and ‘Professional activities’ (13.5%). In contrast, the majority of individuals in our data (based on Latvian tax records) worked in sectors such as ‘Water supply’ (28.3%), ‘Information’ (16.8%), and ‘Arts’ (9.7%) during the same reference period. Therefore, the VT programme seems to mainly target unemployed individuals without any job experience or with experience as employees.

More generally, Latvia is the only Baltic country that registered a decline in self- employment over time, accounting for less than 10% of total employment (Hazans 2005).

Finally, another possibility is that after completing the programme, unemployed youths may seek employment in neighbouring countries, i.e. they may commute or migrate. If they were commuting, we would observe them as employed in another country according to Latvian tax data. This would negatively bias the impact of the programme. In Eastern countries, migration and commuting are quite important. To get a sense of the importance of this issue in our analysis, we run the analysis excluding from our sample individuals who reside in municipalities that share a border with Estonia, Belarus, Russia, or Lithuania. The results are not different from the baseline ones where we consider all municipalities.^40^

### 4: Additional estimation results

**Table 11:** Results using inverse propensity weighting

Employment		Jun. 2016			Dec. 2016			Jul. 2017	
Age	21–29	22–28	23–27	21–29	22–28	23–27	21–29	22–28	23–27
OLS									
Treated	− 0.022	− 0.017	− 0.015	− 0.017	− 0.036*	− 0.036	− 0.036	− 0.022	− 0.037
	(0.021)	(0.024)	(0.029)	(0.021)	(0.021)	(0.024)	(0.024)	(0.029)	(0.029)
Reduced form									
Age< 25	0.007	0.016	0.019	0.003	− 0.000	− 0.001	0.012	0.020	0.043
	(0.024)	(0.027)	(0.033)	(0.024)	(0.024)	(0.027)	(0.027)	(0.033)	(0.033)
2SLS									
Treated	0.059	0.027	− 0.002	0.138	− 0.005	0.099	0.191	0.206	0.432
	(0.194)	(0.191)	(0.194)	(0.234)	(0.229)	(0.232)	(0.337)	(0.331)	(0.342)
*F*-stat	162	162	162	115	115	115	56.8	56.8	56.8
*N*. obs.	9620	9620	9620	7488	7488	7488	5140	5140	5140
Income		Jun. 2016			Dec. 2016			Jul. 2017	
Age	21–29	22–28	23–27	21–29	22–28	23–27	21–29	22–28	23–27
OLS									
Treated	− 59.067***	− 53.442***	− 55.159***	− 67.075***	− 78.273***	− 68.248***	− 62.076***	− 71.081***	− 61.962***
	(13.664)	(15.300)	(16.106)	(15.681)	(17.217)	(18.042)	(19.422)	(20.705)	(22.606)
Reduced form									
Age< 25	1.066	9.884	− 1.828	5.922	8.556	9.883	6.833	20.894	19.153
	(16.399)	(17.028)	(18.698)	(18.769)	(19.464)	(21.023)	(22.515)	(22.844)	(25.034)
2SLS									
Treated	8.661	80.289	− 14.845	50.806	73.404	84.783	68.804	210.383	192.847
	(133.051)	(138.596)	(151.604)	(160.987)	(167.227)	(180.707)	(226.571)	(232.573)	(254.220)
*F*-stat	162	162	162	115	115	115	56.8	56.8	56.8
*N*. obs.	9620	9620	9620	7488	7488	7488	5140	5140	5140

Significance: * *p* < 0.1; ** *p* < 0.05; *** *p* < 0.01

This table shows the reduced form and 2SLS results for the labour market outcomes (employment probability and income) in June 2016, December 2016, and June 2017. We use inverse probability weighting and logistic regressions to estimate the probability of being treated. The predicted probability is then used as a weight in the regression estimations. As in the main analysis, we use a quadratic specification for the polynomial in age. The columns show the results for different age bandwidths: 21–29, 22–28, 23–27. We use the same set of covariates as those reported in the first-stage regressions. Standard errors are in parentheses

## Footnotes

1 Eurostat, Employment and unemployment: Labour Force Survey, 2017. http://ec.europa.eu/eurostat/data/database.

2 National YG schemes are presented to the European Council for approval, and the European Commission monitors their implementation. Updates on the YG in each country are regularly published on the official webpage of the European Commission’s Directorate General for Employment and Social Affairs.

3 Countries that had at least one region with a youth unemployment rate higher than 25% in 2012 were eligible to use funds from the YEI. It was topped up in 2017 for regions with youth unemployment higher than 25% in 2016. The initial 2014–2015 budget was 6.4 billion euros; however, in September 2016, given the still high levels of youth unemployment, the European Commission proposed increasing this budget. In June 2017, the Council and the European Parliament agreed to increase the YEI by 2.4 billion euros for eligible member states for the period of 2017–2020. See http://ec.europa.eu/social/main.jsp?catId=1176.

4 However, several studies have investigated the impact of similar programmes targeting unemployed youth in other EU countries (see Pastore and Zimmermann 2019b and Pastore and Zimmermann 2019a for a collection of empirical studies and Ghirelli et al.^2019^ for the evaluation of a programme targeted at unemployed university graduates in Italy).

5 In Latvian, the programme is known as ‘JG Profesionalas apmacibu programmas’ and is one of the professional training programmes provided by the Latvian Public Employment Service.

6 Following the first version of this paper (Bratti et al. 2018), other studies have evaluated programmes targeting youths and financed with EU funds. For example, Tomić and Zilic (2020) study the impact of an ALMP reform that was implemented in Croatia in 2012, before the YG 2014–2020, to improve the labour market entry of youths without relevant work experience. The programme was initially financed by the Croatian government but later was supported by the YG scheme. Using survey data, they find that the reform had, at best, neutral effects on employment and unemployment. Hora and Sirovátka (2020) analyse an apprenticeship programme implemented under the YG in the Czech Republic. They show that this had a positive impact on the outflow from unemployment for long-term unemployed and medium-skilled youths, but weak effects for the short-term and medium-term unemployed, as well as for low-skilled and high-skilled youth. On this, Cahuc et al. (2013) warned about the danger that the YG would lead to disappointment, given the programme’s ambitious objectives. The authors claimed that extensive public employment and training programmes are not necessarily the perfect solution to create sustainable employment prospects for the unemployed. They suggest that these programmes would be more effective if combined with structural reforms that enhance youth integration in the labour market. In this respect, Germany serves as a good example, with a dual apprenticeship system which is effective in smoothing school-to-work transitions.

7 However, the authors show that after accounting for gender-specific selection effects and the effects of the programmes on pregnancies, gender differences (almost) disappear.

8 The Job Corps evaluations were based on the random assignment of eligible applicants to the programme taking place from the mid-to-late 1990s.

9 To overcome this limitation, Bargain and Doorley (2013) combine an RDD with a structural model to estimate the effect of social transfers for inactive youth in France. They exploit the eligibility age threshold of 25 years with an additional exclusion restriction to be able to make predictions for participation responses at ages further away from the threshold.

10 See, for instance, Ule and Leskošek (2018) for an assessment of Youth Guarantee policies in Slovenia, the Czech Republic, and Latvia; Tosun et al. (2017) for a discussion on youth-oriented ALMPs implemented in the Nordic and the Baltic states; and O’Higgins et al. (2001) for Bulgaria.

11 For instance, an evaluation of a large randomised training programme in Colombia in the early 2000s finds positive effects on employment and earnings (Attanasio et al. 2017; Attanasio et al. 2011). Similar findings emerge from another large-scale experimental training programme in the Dominican Republic, whose short-term effects on employment and earnings have been evaluated by Card et al. (2011), and the long-term effects by Ibarrarán et al. (2014) and Ibarrarán et al. (2015). Results are heterogeneous, with the programmes proving particularly effective for men and young people living in the capital (Ibarrarán et al. 2015). The effects tend to grow over time, confirming that measuring outcomes over a long-enough timespan is crucial.

12 That is, vocational education as such may not be enough. A study in Turkey (Torun and Tumen 2019), for instance, shows that for an individual who pursues a high school degree and does not aim for college education, vocational education does not increase employment prospects relative to general education.

13 Of course, another important argument could be that on-the-job training provides better job-specific training than classroom training.

14 Additional programmes in the YG scheme include (i) measures and workshops offered to young people with sufficient skills but without work experience; (ii) subsidised work offered to disadvantaged young people, such as those with disabilities or the long-term unemployed; and (iii) support for entrepreneurship, offered to young people with the appropriate education or entrepreneurial skills.

15 It is worth mentioning that in 2011, Latvia introduced a large reform of the training system, aiming to make it more competitive through the use of vouchers.

16 The voucher contained information on the maximum amount of expenses covered by the SEA.

17 The list of certified training providers was published on the official webpage of the SEA. The applicant could also select training providers that did not appear in the list if they were ready to make an agreement with the unemployed person and follow the regular procurement procedure. Note that Perez-Johnson et al. (2011) found that it may be suboptimal to give the voucher without any assistance in finding a suitable course.

18 Overall evaluations reported by the training centres point to a high level of satisfaction with these courses. Such documents are held by the Latvian authorities.

19 Unfortunately, the relatively low number of participants does not allow us to investigate heterogeneous effects of the programme by qualification category.

20 Their shares of participants are 25.61%, 23.27%, and 23.72%, respectively.

21 The SRS database collects monthly reports from employers, who declare employees’ monthly incomes (in euros), insurance, working hours, type of occupation based on the International Standard Classification of Occupations (ISCO-88), firm sector (Statistical Classification of Economic Activities in the European Community, i.e. NACE category), and firm size (number of employees).

22 The latest data refer to June 2017.

23 A total of 13,473 registered before December 12, 2013, and 11,900 registered after January 1, 2015.

24 As a robustness check, we also restrict the treatment period to 6 months (86% of treated individuals), and the findings are in line with our baseline results (see Section 6.3).

25 According to the YG guidelines, eligible individuals should be offered participation in the VT (or in any other programme within the YG) within 4 months of registration. From our data, we see that this condition is not always respected as only 65% of the treated group started the training within the first 4 months after registration, with some of them entering the programme 1 year after registration (see Fig. 6 in Appendix A).

26 We can compute the exact age in years starting from age in days.

27 As a sensitivity check, we estimate the same model by allowing the polynomial in the running variable to have different slopes on both sides of the cut-off, as explained in Angrist and Pischke (2009) (pages 197–198). The results do not change (available upon request). We also estimate the same model with a linear polynomial, and the results are robust (available upon request).

28 If the individual is not working, we impute a monthly income equal to 0.

29 For the effect in December 2017 and in the 22–28 bandwidth, the coefficient turns negative. December outcomes may, however, be more sensitive to employment seasonality.

30 See Angrist (2004) and Angrist and Pischke (2009) for the methodology.

31 Our data show that about 62% of the individuals in our sample have Latvian citizenship. The remainder is composed of individuals of Russian nationality (17%) as well as citizens of neighbouring countries, and is unspecified for 15% of cases.

32 Past income is measured in the months of January 2012, June 2012, December 2012, June 2013, and December 2013.

33 These results are available upon request

34 We also estimate the model using non-parametric RDD models using rdrobust (Calonico et al. 2018) but again do not obtain statistically significant results.

35 As an additional robustness check, we apply coarsened exact matching (Iacus et al. 2012), which is a different procedure and applicable if the size of the control group is large and when there are few observables (as in our case). Again, results are not statistically significant and are comparable to those obtained with PSM.

36 https://ec.europa.eu/social/main.jsp?catId=1079&langId=en
